# Antenatal care attendance and risk of low birthweight in Burkina Faso: a cross-sectional study

**DOI:** 10.1186/s12884-021-04310-6

**Published:** 2021-12-13

**Authors:** Mamadou Bountogo, Ali Sié, Alphonse Zakané, Guillaume Compaoré, Thierry Ouédraogo, Elodie Lebas, Jessica Brogdon, Fanice Nyatigo, Benjamin F. Arnold, Thomas M. Lietman, Catherine E. Oldenburg

**Affiliations:** 1 Centre de Recherche en Santé de Nouna, Nouna, Burkina Faso; 2 Francis I Proctor Foundation, University of California, 490 Illinois St, Floor 2, San Francisco, CA 94143 USA; 3 Department of Ophthalmology, University of California, San Francisco, USA; 4 Department of Epidemiology and Biostatistics, University of California, San Francisco, USA

**Keywords:** Antenatal care, Low birthweight, Burkina Faso, Neonates

## Abstract

**Background:**

Low birthweight is a major contributor to infant mortality. We evaluated the association between antenatal care (ANC) attendance and low birthweight among newborns in 5 regions of Burkina Faso.

**Methods:**

We utilized data from the baseline assessment of a randomized controlled trial evaluating azithromycin distribution during the neonatal period for prevention of infant mortality. Neonates were eligible for the trial if the weighed at least 2500 g at enrollment and were 8–27 days of age. Data on ANC attendance and birthweight was extracted from each child’s *carnet de santé*, a government-issued health card on which pregnancy and birth-related data are recorded. We used linear and logistic regression models adjusting for potentially confounding variables to evaluate the relationship between ANC attendance (as total number of visits and ≥ 4 antenatal care visits) and birthweight (continuously and categorized into < 2500 g versus ≥2500 g).

**Results:**

Data from 21,223 births were included in the analysis. The median number of ANC visits was 4 (interquartile range 3 to 5) and 69% of mothers attended at least 4 visits. Mean birthweight was 2998 g (standard deviation 423) and 8.1% of infants were low birthweight (< 2500 g). Birthweight was 63 g (95% CI 46 to 81 g, *P* < 0.001) higher in newborns born to mothers who had attended ≥4 ANC visits versus < 4 visits. The odds of low birthweight among infants born to mothers with ≥4 ANC visits was 0.71 (95% CI 0.63 to 0.79, *P* < 0.001) times the odds of low birthweight among infants born to mothers who attended < 4 ANC visits.

**Conclusions:**

We observed a statistically significant association between ANC attendance and birthweight, although absolute differences were small. Improving access to ANC for all women may help improve birth outcomes.

**Trial registration:**

The parent trial is registered at clinicaltrials.gov: NCT03682653; first registered 24 September 2018.

**Supplementary Information:**

The online version contains supplementary material available at 10.1186/s12884-021-04310-6.

## Background

Reducing the number of infants born with weight < 2500 g by 30% is a key goal of the 2025 Global Nutrition Targets [1]. Low birthweight babies are at increased risk of mortality, morbidity, and poor developmental outcomes compared to normal birthweight babies [2–4]. Low birthweight can occur due to preterm birth or due to restricted intrauterine growth in a full-term infant. Maternal socioeconomic status, including literacy and education, has been previously shown to be associated with low birthweight in low- and middle-income countries [5]. Women with more education may have greater health literacy, access to more resources, and better nutritional status than women without access to education, all of which may influence health care seeking behavior and birth outcomes.

Access to adequate antenatal care services is an important determinant of birth outcomes, and interventions designed to increase antenatal care usage have improved birth outcomes. Until 2016, the World Health Organization recommended receiving antenatal care at least four times during pregnancy, which was revised to 8 visits in 2016 [6]. Essential components of antenatal care, such as folic acid supplementation, nutritional advice, and screening for infectious disease and conditions such as pre-eclampsia, may improve intrauterine growth and neonatal survival. Maternal malaria infection and undernutrition have been shown to increase risk of low birthweight [7]. Intermittent preventive treatment (IPT) for malaria is delivered as part of antenatal care during the second and third trimesters in malaria-endemic areas [8, 9]. Inadequate antenatal care attendance can limit the number of doses of IPT delivered and result on worse birth outcomes due to malaria [9].

We evaluated the relationship between antenatal care attendance and birthweight in a large study of neonatal azithromycin administration for prevention of infant mortality in 5 regions of Burkina Faso. Burkina Faso is a landlocked country in the West African Sahel that experiences highly seasonal rainfall that corresponds with a high malaria transmission season and high malnutrition season [10]. We hypothesized that birthweight would be higher in women with a great number of antenatal care visits.

## Methods

We utilized data from the baseline assessment of a randomized controlled trial evaluating whether a single oral dose of azithromycin administered during the neonatal period was effective for reducing infant mortality in Burkina Faso (clinicaltrials.gov NCT03682653) [11]. The trial was conducted in 44 primary healthcare facilities in 5 regions of Burkina Faso, including Centre, Boucle du Mouhoun, Cascade, Centre Ouest, and Hauts-Bassins. Enrollment lasted from April 2019 through December 2020. Facilities were in a mix of urban and rural settings. Because the parent trial was comparing oral azithromycin administered in the neonatal period to placebo, facilities were chosen to be within 4 h of a pediatric hospital that had pediatric surgical facilities in the case of infantile hypertrophic pyloric stenosis, a rare but serious condition that has been linked to azithromycin administration in observational studies [12]. Neonates were eligible for the trial if they were between 8 and 27 days of age, weighed at least 2500 g at enrollment, were able to feed orally, and did not have clinical signs of neonatal jaundice. Low birthweight babies were not excluded from the trial, but the child had to have gained enough weight to meet the trial’s weight-based enrollment criterion (2500 g) by the time they were 27 days of age to be able to participate in the parent trial and be included in this analysis. Only infants with birthweights recorded in the *carnet de santé* (government-issued health card) with complete data on antenatal care attendance were included in the present analysis. Neonates were recruited via contacting mothers who gave birth in the facilities and outreach during BCG vaccination days. The Institutional Review Boards at the University of California, San Francisco and the Comité National d’Ethique pour la Recherche (National Research Ethics Committee) in Ouagadougou, Burkina Faso reviewed and approved the study. Written informed consent was provided by the caregiver of each neonate enrolled in the trial. All study procedures were carried out in accordance with the Declaration of Helsinki and relevant guidelines and regulations.

At enrollment, a baseline questionnaire was completed by the caregiver of each enrolled neonate. The questionnaire included the child’s age at enrollment and sex, maternal age and education (coded as none, primary, secondary, or higher than secondary school), and the number of times the mother had been pregnant. Questions related to the pregnancy included whether the child was a singleton or multiple birth and if the mother had given birth in a healthcare facility. We extracted data from the child’s health and vaccination card (*carnet de santé*) on the number of antenatal clinic visits attended by the mother and the neonate’s birthweight. We collected data on each healthcare facility included in the study, including whether it was in an urban or rural setting, if the facility has a physician on-site, and the number of non-physician clinicians, including nurses and midwives, employed by the facility. Because infants were not enrolled during pregnancy and gestational age testing is not widely available in the study area, information on gestational age was not collected.

We evaluated the relationship between the number of antenatal care visits and 1) birthweight as a continuous variable using linear regression models and 2) low birthweight (defined as < 2500 g) using logistic regression models. All multivariable models were adjusted for maternal age, education, number of previous pregnancies, type of pregnancy (singleton vs multiple), the infant’s sex, region of the facility, if the facility was in an urban or rural setting, and if it had a physician onsite (as a proxy for level of care available at the facility). These variables were chosen because they were hypothesized to be confounders of the relationship between antenatal care attendance and birthweight. We chose this strategy as other strategies that rely on statistical criteria or comparison of adjusted and unadjusted effects may lead to omission of important but non-statistically significant confounders from the analysis and inappropriate adjustment for variables that are on the causal pathway between exposure and outcome [13]. We then evaluated the same outcomes using whether the mother had attended at least four antenatal care visits, adjusting for the same potentially confounding variables. We first ran a series of univariate models for antenatal care use and each confounding variable, and then a multivariable model for each exposure and outcome pair including all covariates. We did not base confounding decisions on statistical significance in the univariate model, due to known biases arising from relying on statistical criteria for confounder selection [14]. For all exposure-outcome pairs, we conducted a subgroup analysis among rural versus urban facilities to evaluate any differences the association between antenatal care use and birthweight by urbanicity. Due to the multilevel nature of the data, all models adjusted for clustering at the facility level using a Huber-White sandwich estimator. All analyses were conducted in Stata version 15.1 (StataCorp, College Station, TX).

## Results

Of 21,832 neonates enrolled in the parent study, 21,319 (97.7%) had valid birthweight measurements recorded in the *carnet de santé* and 21,223 (97.3%) had complete data on antenatal care and the healthcare facility and were included in the analysis. The mothers of most (*N* = 21,166, 99.7%) infants had attended at least one antenatal care visit during pregnancy, and 14,601 (68.8%) attended at least four. Only 3 women (0.01%) had 8 antenatal care visits, and no women attended more than 8 visits. Mean birthweight was 2998 g (standard deviation, SD, 423 g) and 8.1% of the enrolled population was low birthweight (Table 1). Female infants had lower mean birthweights and were more often low birthweight than male infants (females: mean birthweight 2942 g, SD 407, 9.6% low birthweight; males: mean birthweight 3053 g, SD 432, 6.6% low birthweight; *P* < 0.001 for both comparisons). Although twins made up only a small percentage of enrolled, participants, they had much lower birthweights (twins: mean birthweight 2554 g, SD 335; singletons: mean birthweight 3006 g, SD 421; *P* < 0.001) and were much more likely to be low birthweight (39.2% of twins were low birthweight versus 7.5% of singletons; *P* < 0.001).

**Table 1:** Descriptive characteristics for study sample, overall and by birthweight status (low birthweight/normal birthweight)

	Overall	Low birthweight (< 2500 g)	Normal birthweight (≥2500 g)	Mean Birthweight, g (SD)
N	21,223	1715 (8.1%)	19,508 (91.9%)	2998 (423)
Child’s sex				
Male	10,685 (50.3%)	701 (40.9%)	9984 (51.2%)	3053 (432)
Female	10,538 (49.7%)	1014 (59.1%)	9524 (48.8%)	2942 (407)
Antenatal care visits, median (IQR)	4 (3 to 5)	4 (3 to 4)	4 (3 to 5)	N/A
≥ 4 antenatal care visits	14,601 (68.8%)	1056 (61.6%)	13,545 (69.4%)	3018 (424)
Type of pregnancy				
Singleton	20,863 (98.3%)	1574 (91.8%)	19,289 (98.9%)	3006 (421)
Multiple	360 (1.7%)	141 (8.2%)	219 (1.1%)	2554 (335)
Region				
Centre	1803 (8.5%)	191 (11.1%)	1612 (8.3%)	2937 (409)
Boucle du Mouhoun	2536 (12.0%)	242 (14.1%)	2294 (11.8%)	2973 (426)
Cascade	3936 (18.5%)	261 (15.2%)	3675 (18.8%)	3030 (432)
Centre Ouest	2425 (11.4%)	195 (11.4%)	2230 (11.4%)	2975 (419)
Hauts-Bassins	10,523 (49.6%)	826 (48.2%)	9697 (49.7%)	3008 (422)
Setting				
Urban	17,736 (83.6%)	1401 (81.7%)	16,335 (83.7%)	2986 (420)
Rural	3487 (16.4%)	314 (18.3%)	3173 (16.3%)	3001 (424)
Physician onsite at birth facility	2657 (12.5%)	206 (12.1%)	2451 (12.6%)	3009 (428)
Maternal age, years, mean (SD)	26.1 (6.2)	24.7 (6.2)	26.2 (6.2)	N/A
Maternal education				
None	11,542 (54.4%)	956 (55.7%)	10,586 (54.3%)	2999 (422)
Primary	3867 (18.2%)	286 (16.7%)	3581 (18.3%)	3000 (420)
Secondary	5208 (24.5%)	430 (25.0%)	4779 (24.5%)	2991 (426)
Higher than secondary	606 (2.9%)	44 (2.6%)	562 (2.9%)	3027 (442)
Previous gravidity, median (IQR)	2 (0 to 3)	1 (0 to 2)	2 (0 to 3)	N/A

Among infants whose mothers attended at least 4 antenatal care visits, mean birthweight was 3018 g (SD 424) versus 2954 g (SD 419) among those who attended < 4 antenatal care visits, and 7.2% of infants born to mothers who attended at least 4 antenatal care visits were low birthweight compared to 9.9% of those who attended < 4 (Table 1). In models adjusted for pregnancy, maternal, and facility characteristics, each additional antenatal care visit was associated with a 31 g increase in birthweight (95% confidence interval, CI, 23 to 39 g, *P* < 0.001; Table 2). Each additional antenatal care visit was associated with 0.85 times the odds of low birthweight (95% CI 0.81 to 0.89, *P* < 0.001; Table 3). In models stratified by urban versus rural setting, the relationship between antenatal care attendance and birthweight was strongest in babies enrolled in urban facilities, with no evidence of a difference in birthweight by number of antenatal care visits among rural-dwelling neonates (Supplemental Tables 1 and 2).

**Table 2:** Associations between birthweight and number of antenatal care visits (*N* = 21,223)

	Univariate		Multivariable	
	Mean Difference, g (95% CI)	*P*-value	Adjusted Mean Difference, g (95% CI)	*P*-value
No. of ANC visits	31 (23 to 40)	< 0.001	31 (23 to 39)	< 0.001
Maternal age, per year	11 (10 to 12)	< 0.001	5 (3 to 7)	< 0.001
Maternal education				
None	Ref		Ref	
Primary	1 (−21 to 23)	0.94	23 (4 to 42)	0.02
Secondary	−8 (−27 to 10)	0.37	36 (23 to 49)	< 0.001
Higher than secondary	28 (−27 to 82)	0.31	62 (18 to 107)	0.008
Gravidity, per pregnancy	37 (32 to 43)	< 0.001	31 (23 to 39)	< 0.001
Pregnancy type				
Singleton	Ref		Ref	
Multiple	− 452 (− 506 to − 398)	< 0.001	− 473 (− 525 to −421)	< 0.001
Infant’s sex				
Male	Ref		Ref	
Female	− 111 (− 122 to − 100)	< 0.001	− 112 (− 123 to − 101)	< 0.001
Facility setting				
Urban	Ref		Ref	
Rural	−15 (−83 to 52)	0.65	−23 (− 96 to 50)	0.52
Physician onsite				
No	Ref		Ref	
Yes	12 (−21 to 46)	0.47	16 (− 34 to 66)	0.53
Region				
Centre	Ref		Ref	
Boucle du Mouhoun	37 (−25 to 98)	0.23	43 (−26 to 112)	0.21
Cascade	93 (50 to 137)	< 0.001	83 (32 to 135)	0.002
Centre Ouest	38 (−18 to 93)	0.18	23 (−38 to 85)	0.45
Hauts-Bassins	71 (27 to 115)	0.002	81 (34 to 127)	0.001

**Table 3:** Associations between low birthweight (< 2500 g) and number of antenatal care visits (*N* = 21,223)

	Univariate		Multivariable	
	Odds Ratio (95% CI)	*P*-value	Adjusted Odds Ratio (95% CI)	*P*-value
No. of ANC visits	0.85 (0.81 to 0.90)	< 0.001	0.85 (0.81 to 0.89)	< 0.001
Maternal age, per year	0.96 (0.95 to 0.97)	< 0.001	0.98 (0.97 to 1.00)	0.02
Maternal education				
None	Ref		Ref	
Primary	0.88 (0.74 to 1.05)	0.16	0.82 (0.69 to 0.98)	0.03
Secondary	0.99 (0.87 to 1.14)	0.93	0.87 (0.76 to 1.00)	0.06
Higher than secondary	0.87 (0.67 to 1.12)	0.27	0.79 (0.59 to 1.05)	0.11
Gravidity, per pregnancy	0.86 (0.82 to 0.90)	< 0.001	0.86 (0.80 to 0.92)	< 0.001
Pregnancy type				
Singleton	Ref		Ref	
Multiple	7.89 (5.70 to 10.92)	< 0.001	9.20 (6.69 to 12.65)	< 0.001
Infant’s sex				
Male	Ref		Ref	
Female	1.52 (1.36 to 1.69)	< 0.001	1.56 (1.41 to 1.73)	< 0.001
Facility setting				
Urban	Ref		Ref	
Rural	1.15 (0.87 to 1.53)	0.32	1.16 (0.83 to 1.64)	0.38
Physician onsite				
No	Ref		Ref	
Yes	0.95 (0.78 to 1.16)	0.61	0.96 (0.78 to 1.18)	0.70
Region				
Centre	Ref		Ref	
Boucle du Mouhoun	0.89 (0.68 to 1.17)	0.41	0.87 (0.65 to 1.15)	0.32
Cascade	0.60 (0.46 to 0.78)	< 0.001	0.62 (0.49 to 0.77)	< 0.001
Centre Ouest	0.74 (0.57 to 0.95)	0.02	0.80 (0.63 to 1.03)	0.08
Hauts-Bassins	0.72 (0.56 to 0.93)	0.01	0.68 (0.57 to 0.84)	< 0.001

Results were similar in models categorizing the number of antenatal care visits (≥4 versus < 4, Table 4).

**Table 4:** Multivariable associations between birthweight and low birthweight (< 2500 g) and ≥ 4 antenatal care visits (*N* = 21,223)

	Birthweight (g)		Low birthweight (< 2500 g)	
	Mean Difference, g (95% CI)	*P*-value	Mean Difference, g (95% CI)	*P*-value
≥4 ANC visits, univariate^a^	63 (46 to 81)	< 0.001	0.71 (0.63 to 0.79)	< 0.001
≥4 ANC visits	61 (45 to 76)	< 0.001	31 (23 to 39)	< 0.001
Maternal age, per year	5 (4 to 7)	< 0.001	5 (3 to 7)	< 0.001
Maternal education				
None	Ref		Ref	
Primary	23 (5 to 41)	0.02	23 (4 to 42)	0.02
Secondary	37 (24 to 50)	< 0.001	36 (23 to 49)	< 0.001
Higher than secondary	67 (22 to 111)	0.004	62 (18 to 107)	0.008
Gravidity, per pregnancy	30 (23 to 38)	< 0.001	31 (23 to 39)	< 0.001
Pregnancy type				
Singleton	Ref		Ref	
Multiple	− 470 (− 522 to 419)	< 0.001	−473 (−525 to −421)	< 0.001
Infant’s sex				
Male	Ref		Ref	
Female	−111 (−122 to −101)	< 0.001	−112 (−123 to − 101)	< 0.001
Facility setting				
Urban	Ref		Ref	
Rural	−23 (−95 to 49)	0.53	−23 (−96 to 50)	0.52
Physician onsite				
No	Ref		Ref	
Yes	17 (−32 to 66)	0.49	16 (−34 to 66)	0.53
Region				
Centre	Ref		Ref	
Boucle du Mouhoun	43 (−26 to 112)	0.22	43 (−26 to 112)	0.21
Cascade	86 (0.35 to 137)	0.002	83 (32 to 135)	0.002
Centre Ouest	25 (−35 to 84)	0.41	23 (−38 to 85)	0.45
Hauts-Bassins	80 (34 to 126)	0.001	81 (34 to 127)	0.001

^a^Univariate analysis evaluating ≥4 ANC visits predicting birthweight and low birthweight, unadjusted for covariates, with standard errors adjusted for clustering within enrollment facility

## Discussion

In this analysis of more than 21,000 neonates enrolled in 44 primary healthcare facilities across Burkina Faso, we found evidence of a small difference in birthweight among neonates born to mothers who had attended a higher number of antenatal clinic visits. These results are in line with previous evidence from other settings that have documented that major risk factors for low birthweight include poor antenatal care attendance [15, 16]. However, absolute differences observed in the present analysis were fairly small. For example, the risk of low birthweight was higher in the Democratic Republic of Congo, Zambia, Kenya, and Uganda among women with fewer antenatal care visits than in the present analysis [15, 16]. We found similar results treating the number of antenatal care visits continuously or as a dichotomous variable (≥4 visits). Attending at least four antenatal care visits was recommended by the WHO until 2016, and thus we used this cutoff to assess whether “adequate” receipt of antenatal care was associated with birthweight outcomes. Most participants in the present study were urban, and determinants of low birthweight may be different in urban compared to rural settings. Barriers to accessing antenatal care may also be different in urban settings where factors such as travel times are less of an issue, but long wait times or other structural barriers reduce access to services [17]. In stratified models, we found that the relationship between antenatal care attendance and low birthweight was predominantly driven by babies enrolled in urban settings, and we found no evidence of a difference in birthweight outcomes by antenatal care attendance among those born in rural settings, despite an overall higher proportion of rural-dwelling babies born at low birthweight and a lower antenatal care attendance among rural-dwelling mothers. This may indicate that the relationship between antenatal care use and birthweight is confounded by another factor, such as socioeconomic status, in urban but not rural populations, or that there are systematic differences in the impact of antenatal care on birth outcomes in urban compared to rural women.

The vast majority of participants in this analysis reported at least one antenatal care visit. This may have been due to sampling at primary healthcare facilities where infants were born, which primary reached infants who had been born in the facility and may have been more likely to have had antenatal care services compared to sampling in communities. Home births are relatively uncommon in Burkina Faso, and there are no fees for antenatal care or facility delivery, which may improve access to both [18, 19]. Previous analyses have demonstrated high use of antenatal care in Burkina Faso [6, 20]. However, in the present analysis only two-thirds of participants attended four or more antenatal care visits, in line with previous estimates from West Africa [6]. Almost none of the mothers in our analysis attended 8 or more antenatal care visits, suggesting that there are major gaps between antenatal care use and current WHO recommendations.

This analysis must be considered in the context of several limitations. Infants included in the parent trial had to be at least 2500 g at enrollment and were at least 8 days of age, meaning that the analysis was restricted to those who survived long enough and who weighed enough to be able to participate in the parent trial. If antenatal care attendance is associated with survival, this may have induced selection bias for several reasons: 1) babies were less likely to be low birthweight due to the weight criterion than those in the general populations; 2) babies who did not survive their first week of life were not included; and 3) low birthweight babies would have had to gain weight quickly to be eligible to be included in the parent trial. We were not able to estimate gestational age, so we cannot comment on differences between babies who were preterm or if low birthweight was due to restricted intrauterine growth. Previous studies have suggested that quality of antenatal care is as important as quantity of antenatal care. We did not collect data on quality of antenatal care and relied on routinely recorded data in the mother’s health care, so are unable to comment on the quality of care. However, we collected healthcare facility-level data that may serve as a proxy for quality, although none of these were associated with differences in birthweight or probability of low birthweight. There may have been unmeasured confounding, particularly by socioeconomic status, as our socioeconomic status measurements were limited to mother’s educational attainment and indicators for whether the facility was in a rural or urban setting. Women with higher socioeconomic status may have improved access to antenatal care and independently higher birthweight babies due to improved access to nutrition and other resources. Although we enrolled a large population across diverse geographic settings in Burkina Faso, these results may not be generalizable to very rural populations or to very low birthweight babies.

## Conclusions

In this large sample of neonates enrolled across diverse settings in Burkina Faso, we found a significant association between increased antenatal care attendance and birthweight. Babies born to mothers who had attended at least 4 antenatal care visits had higher mean birthweights and were less likely to be born low birthweight. Increasing access to adequate antenatal care may be a useful component of improving birth outcomes in Burkina Faso.

## Supplementary Information

**Additional file 1: Supplemental Table 1.** Association between number of antenatal care visits and birthweight among urban- (*N* = 17,736) and rural-dwelling (*N* = 3487) newborns in 5 regions of Burkina Faso. **Supplemental Table 2.** Association between number of antenatal care visits and low birthweight (< 2500 g) among urban- (*N* = 17,736) and rural-dwelling (*N* = 3487) newborns in 5 regions of Burkina Faso.

## Data Availability

The datasets analyzed in this study are available from the corresponding author upon reasonable request.

## Abbreviations

ANC: Antenatal care
BCG: Bacillus Calmette-Guérin
CI: Confidence interval
IPT: Intermittent preventive treatment
IQR: Interquartile range
SD: Standard deviation
