# Supplementary material for: Antenatal care attendance and risk of low birthweight in Burkina Faso: a cross-sectional study
Source: BMC Pregnancy Childbirth. 2021 Dec 13;21:825. doi: 10.1186/s12884-021-04310-6 (PMC8667364; doi:10.1186/s12884-021-04310-6)
Supplement: Supplementary file 1 — Additional file 1: Supplemental Table 1. Association between number of antenatal care visits and birthweight among urban- (N = 17,736) and rural-dwelling (N = 3487) newborns in 5 regions of Burkina Faso. Supplemental Table 2. Association between number of antenatal care visits and low birthweight (< 2500 g) among urban- (N = 17,736) and rural-dwelling (N = 3487) newborns in 5 regions of Burkina Faso. [file 12884_2021_4310_MOESM1_ESM.docx]

**Supplemental Table 1.** Association between number of antenatal care visits and birthweight among urban- (N=17,736) and rural-dwelling (N=3,487) newborns in 5 regions of Burkina Faso

|  | Urban (N=17,736) | | Rural (N=3,487) | |
| --- | --- | --- | --- | --- |
|  | Adjusted Mean Difference, g  (95% CI) | *P*-value | Adjusted Mean Difference, g  (95% CI) | *P*-value |
| No. of ANC visits | 35 (26 to 44) | <0.001 | 17 (-4 to 37) | 0.10 |
| Maternal age, per year | 4 (3 to 6) | <0.001 | 9 (3 to 14) | 0.007 |
| Maternal education |  |  |  |  |
| None | Ref |  | Ref |  |
| Primary | 14 (-3 to 31) | 0.11 | 59 (-28 to 145) | 0.16 |
| Secondary | 36 (22 to 50) | <0.001 | 26 (-42 to 95) | 0.41 |
| Higher than secondary | 63 (18 to 109) | 0.008 | 15 (-51 to 82) | 0.62 |
| Gravidity, per pregnancy | 35 (27 to 43) | <0.001 | 17 (-2 to 36) | 0.07 |
| Pregnancy type |  |  |  |  |
| Singleton | Ref |  | Ref |  |
| Multiple | -461 (-523 to -399) | <0.001 | -514 (-614 to -414) | <0.001 |
| Infant’s sex |  |  |  |  |
| Male | Ref |  | Ref |  |
| Female | -111 (-123 to -99) | <0.001 | -117 (-145 to -88) | <0.001 |
| Physician onsite |  |  |  |  |
| No | Ref |  | Ref |  |
| Yes | 3 (-41 to 47) | 0.89 | 49 (36 to 63) | <0.001 |
| Region |  |  |  |  |
| Centre | Ref |  | NA^1^ |  |
| Boucle du Mouhoun | 17 (-44 to 77) | 0.58 | Ref |  |
| Cascade | 85 (34 to 136) | 0.002 | -59 (-81 to 38) | <0.001 |
| Centre Ouest | 35 (-28 to 98) | 0.26 | -244 (-264 to -224) | <0.001 |
| Hauts-Bassins | 86 (39 to 133) | 0.001 | -78 (-169 to 13) | 0.08 |

^1^All participants in Centre region were urban-dwelling (Ouagadougou)

**Supplemental Table 2.** Association between number of antenatal care visits and low birthweight (<2500 g) among urban- (N=17,736) and rural-dwelling (N=3,487) newborns in 5 regions of Burkina Faso

|  | Urban (N=17,736) | | Rural (N=3,487) | |
| --- | --- | --- | --- | --- |
|  | Odds Ratio  (95% CI) | *P*-value | Odds Ratio  (95% CI) | *P*-value |
| No. of ANC visits | 0.83 (0.79 to 0.88) | <0.001 | 0.94 (0.85 to 1.03) | 0.20 |
| Maternal age, per year | 0.99 (0.97 to 1.01) | 0.99 | 0.95 (0.93 to 0.98) | <0.001 |
| Maternal education |  |  |  |  |
| None | Ref |  | Ref |  |
| Primary | 0.84 (0.69 to 1.01) | 0.07 | 0.86 (0.58 to 1.29) | 0.47 |
| Secondary | 0.88 (0.75 to 1.03) | 0.10 | 0.72 (0.46 to 1.11) | 0.14 |
| Higher than secondary | 0.77 (0.57 to 1.03) | 0.08 | 2.24 (0.95 to 5.25) | 0.06 |
| Gravidity, per pregnancy | 0.84 (0.78 to 0.90) | <0.001 | 0.95 (0.82 to 1.11) | 0.53 |
| Pregnancy type |  |  |  |  |
| Singleton | Ref |  | Ref |  |
| Multiple | 8.29 (5.80 to 11.86) | <0.001 | 0.95 (0.82 to 1.11) | 0.53 |
| Infant’s sex |  |  |  |  |
| Male | Ref |  | Ref |  |
| Female | 1.59 (1.42 to 1.78) | <0.001 | 14.17 (7.50 to 26.79) | <0.001 |
| Physician onsite |  |  |  |  |
| No | Ref |  | Ref |  |
| Yes | 1.00 (0.82 to 1.23) | 0.89 | 1.44 (1.11 to 1.86) | <0.001 |
| Region |  |  |  |  |
| Centre | Ref |  | NA^1^ |  |
| Boucle du Mouhoun | 0.98 (0.78 to 1.22) | 0.82 | Ref |  |
| Cascade | 0.60 (0.47 to 0.76) | <0.001 | 2.28 (2.05 to 2.54) | <0.001 |
| Centre Ouest | 0.76 (0.59 to 0.99) | 0.04 | 3.64 (3.25 to 4.07) | <0.001 |
| Hauts-Bassins | 0.67 (0.54 to 0.83) | <0.001 | 2.06 (1.36 to 3.11) | 0.001 |

^1^All participants in Centre region were urban-dwelling (Ouagadougou)
